# Network Analysis Shows Novel Molecular Mechanisms of Action for Copper-Based Chemotherapy

**DOI:** 10.3389/fphys.2015.00406

**Published:** 2016-01-12

**Authors:** Jesús Espinal-Enríquez, Enrique Hernández-Lemus, Carmen Mejía, Lena Ruiz-Azuara

**Affiliations:** ^1^Computational Genomics, National Institute of Genomic MedicineMéxico City, Mexico; ^2^Complejidad en Biología de Sistemas, Centro de Ciencias de la Complejidad, Universidad Nacional Autónoma de MéxicoCiudad de México, Mexico; ^3^Facultad de Ciencias Naturales, Universidad Autónoma de QuerétaroQuerétaro, Mexico; ^4^Departamento de Química Inorgánica y Nuclear, Facultad de Química, Universidad Nacional Autónoma de MéxicoCiudad de México, Mexico

**Keywords:** causal network analysis, copper-based chemotherapy, systems pharmacology, Casiopeína II-gly, liver protection

## Abstract

The understanding of the mechanisms associated with the action of chemotherapeutic agents is fundamental to assess and account for possible side-effects of such treatments. Casiopeínas have demonstrated a cytotoxic effect by activation of pro-apoptotic processes in malignant cells. Such processes have been proved to activate the apoptotic intrinsic route, as well as cell cycle arrest. Despite this knowledge, the whole mechanism of action of Casiopeínas is yet to be completely understood. In this work we implement a systems biology approach based on two pathway analysis tools (Over-Representation Analysis and Causal Network Analysis) to observe changes in some hallmarks of cancer, induced by this copper-based chemotherapeutic agent in HeLa cell lines. We find that the metabolism of metal ions is exacerbated, as well as cell division processes being globally diminished. We also show that cellular migration and proliferation events are decreased. Moreover, the molecular mechanisms of liver protection are increased in the cell cultures under the actions of Casiopeínas, unlike the case in many other cytotoxic drugs. We argue that this chemotherapeutic agent may be promising, given its protective hepatic function, concomitant with its cytotoxic participation in the onset of apoptotic processes in malignant cells.

## Introduction

From the molecular and physiological points of view, the set of pathological conditions commonly described as cancer presents a complex phenomenology that influences the design of therapeutic strategies. In particular, the use of anti-cancer pharmacological therapies directed at specific molecular targets is usually considered among the best strategies to alleviate the disease. However, due to the dense and robust network of (abnormal) interactions set-up by the biomolecular and physiological alterations typical of cancer, the efficacy of such targeted therapies is often challenged (de Anda-Jáuregui et al., 2015). In such cases is necessary to resort to less-specific drugs (chemotherapeutics), whose mechanisms of action are mostly cytotoxic. Cytotoxic therapy presents a number of disadvantages when compared with its targeted counterparts. Most cytotoxic compounds are highly unspecific and damaging at the organismal level. For instance, metal-based chemotherapy (such as the alkylating agent cisplatin) presents DNA damaging activity and induces cell death (primarily by apoptosis but also by necrosis) in an uncontrolled way (cisplatin is a cell-cycle unspecific drug). Long term effects in humans include anemia and leukopenia, liver and kidney damage, neurological diseases (such as peripheral neuropathy), serum electrolyte disturbances and vascular toxicity among others.

Research in chemotherapy aims at developing alternative cytotoxic agents to be used as anti-cancer drugs. Such drugs will ideally avoid some of the most harmful side effects of current metal-based chemotherapy. In this regard, copper-based drugs known as Casiopeínas present a series of advantages, such as the fact that copper-compounds may be metabolized, thus reducing indirect toxicity and metal-related poisoning (Ruiz-Azuara, 1997; Bravo-Gómez et al., 2009; Valencia-Cruz et al., 2013). Aside from this, the pharmacological dosage is lower than that of competing therapies.

To evaluate the systemic effect of such novel cytotoxic compounds, an integrative view is desirable since—as we already mentioned—secondary immediate and long-term effects arise from their use. Systems biology provides precisely such view by incorporating elements from high-throughput omic technologies with strong computational and analytical tools. In the past, some systems biology attempts to study the effects of Casiopeínas have been carried out (Gutiérrez et al., 2013; Valencia-Cruz et al., 2013). Those efforts, however, were focused in the anti-proliferative effects (increasing cell death and arresting cell cycle) which are of course central to understand the main mechanisms of antineoplastic action. Other biological processes may, however, be affected by Casiopeínas, so that new molecular functions—that may add to the therapeutic efficacy and possibly to collateral effects—are expected to rise. It is desirable then, to account for these effects in a quantitative/qualitative manner aiming at a mechanistic characterization (Hernández-Lemus, 2013).

Genome-wide gene expression analysis (GEA) provides an appropriate tool to study systems-level biomolecular processes responsible for phenotypic traits. However, GEA alone falls short represent all the complex interactions present among such processes. For this reason, *Pathway Analysis methods* have been developed to improve the predictive power derived from such high-throughput experiments (Tarca et al., 2009; Vaske et al., 2010; Drier et al., 2013; Krämer et al., 2013; Wang et al., 2013; Huang et al., 2014; Verhaegh et al., 2014). Pathway level studies have revealed new insight that may lead to a functional picture of complex phenotypes such as the ones associated with cancer (Lohr et al., 2012). Such methods are built upon system-level analytics, consisting in probabilistic and computational modeling and simulation strategies. Those strategies are performed in large amounts of experimental data contained in carefully curated information repositories, that may be in the form of structured *Knowledge-based* data bases, but also in unstructured *data-mining* and *literature-search* applications.

To date, a vast majority of the pathway analysis tools is based on statistical tests on the number of differentially expressed genes that are enlisted as part of some biological pathway. These methods are commonly referred to as *enrichment* or *over-representation* methods. These approaches, though very simple, have provided an additional dimension (aimed at a functional description) to the large scale molecular study of biological systems. The analysis of cellular processes under the perspective of biological interaction networks has revealed foundational principles; these in turn may lead to the formulation of functional and mechanistic hypotheses to be further tested and refined. In this regard, knowledge-based sources such as Ingenuity Knowledge-Base (IKB, Krämer et al., 2013) present an effective alternative (based on highly curated information sources, relying on detailed experimental evidence and extensive literature references) to large scale probabilistic and computational modeling, especially useful when facing small sample counts, as is the case here.

## Materials and methods

### Synthesis of Casiopeína II gly

Copper complexes, Casiopeína II-gly (Cas II-gly), were synthesized by starting with an equimolar solution of copper (II) nitrate mixed together with the corresponding substituted diimine (in this case, glycine), followed by the addition of a N-O donor previously deprotonated, as previously reported (Bravo-Gómez et al., 2009). Infrared (IR) spectrum analysis followed synthesis and the product was compliant with a previous characterization (Ruiz-Azuara, 1997).

### Cell line cultures

The HeLa cell line (American Type Culture Collection, CCL-2; Rockville, MD, USA) was maintained at 37°C in 5% CO_2_ under sterile conditions in Dulbecco's modified Eagle medium (DMEM, Sigma Chemical Co., St Louis, MO, USA) supplemented with 10% fetal bovine serum (Sigma) and treated for 6 h with IC_50_ of Cas II-gly (40 μM) in 96 microplates (Valencia-Cruz et al., 2013). Cells were stained with sulphorrodamine-B and absorbance was quantified in a spectrophotometer at 560 nm (Lab-system Uniskan, Manchester, UK).

### Cellular viability

Cellular viability was estimated by the 3-(4,5-dimethythiazol-2y1)-2,5-diphenyl-tetrazolium bromide (MTT) assay (Roche Diagnostics, Mannheim, Germany). Briefly, cells (2 × 10^5^ per well) were seeded in 24-well culture plates and preincubated for 24 h. After exposure to treatment for 24 h at 37°C, 2 ml of MTT (0.1 *mg*∕*ml*) was added, and cells were then incubated for 3 h at 37°C in darkness. The tetrazolium crystals were solubilized by the addition of 10% SDS in 0.01 N HCl, and the formazan blue formed from MTT was quantified with a spectrophotometer at 560 nm (Lab-system, Uniskan, Manchester, UK).

### RNA extraction and preparation

In order to extract RNA, HeLa cells (5 × 10^6^ cells) treated or not with 40 μM Cas II-gly for 6 h were collected in 1 ml of Trizol (Invitrogen) and extraction was performed according to manufacturer's instructions. RNA integrity was determined by means of gel electrophoresis, and nucleic acid concentration was measured with a spectrophotometer (Amersham Pharmacia Biotech). Reverse transcription was performed with Superscript II Reverse Transcriptase (Invitrogen, No. cat.18064-014) and cDNA was amplified with Taq DNA Polymerase (Invitrogen, No. cat. 11615-010). PolyA+ RNA from HeLa cells were selected by affinity chromatography using an oligo (dT) cellulose column.

### Genome-wide microarray gene expression analysis

Genome wide gene expression experiments were performed in a set of double-triplicates for cases (treatment with Cas II-gly) and controls. Total mRNA was extracted from the two sets of cells and processes under the GPL570 protocol in Affymetrix HGU133Plus2 arrays (Valencia-Cruz et al., 2013). Gene expression data has been publicly archived at NCBI GEO under accession key GSE41827. Additional information can be also found as a NCBI BioProject with Accession PRJNA178551 and ID 178551.

### Pathway enrichment analysis

Web-GESTALT (Wang et al., 2013) enrichment tool is focused mainly in the identification of over-represented pathways with the rationale that the proportion of differential expressed genes, within a given pathway, exceeds the proportion of genes that could be randomly expected (Garcia-Campos et al., 2015). Pathway enrichment was performed in order to uncover enriched pathways compliant with the set of differentially expressed genes obtained by comparing the HeLa cell lines treated with Cas-II gly vs. a control group. Web-GESTALT is a comprehensive large scale data-mining webtool. It covers 78,612 functional categories over a dozen of large scale public databases. It performs statistical enrichment over the full set of annotated pathways and calculates statistical significance by means of FDR-corrected hypergeometric tests. In the work presented here, we used a corrected *p*-value threshold of 10^−4^.

### Causal network analysis

In order to validate and complement the results obtained by the pathway enrichment analysis, a causal network analysis was performed. The *Ingenuity Pathway Analysis* toolkit (IPA ®, QIAGEN Redwood City, www.qiagen.com/ingenuity) was then used to generate Causal Networks (Krämer et al., 2013). IPA depends on a highly curated knowledge-based archive known as the *IKB*. IKB integrates data from more than 40,000 nodes representing mammalian genes and their products (transcripts, proteins, miRNAs, second messengers, etc.) in addition to chemical compounds (both exogenous and endogenous). IKB archives more than 1,480,000 interactions between molecules. Such interactions conform the set of links between the nodes which represent *experimentally observed* cause-effect relationships (hence the name, Causal Networks) relative to transcription, expression, activation, molecular modification, binding events, and transport processes. These interactions have been experimentally measured, so they can be associated with a definite direction of the causal effect—activation or inhibition—of the given processes at a whole genome network-wide level.

**Figure 1:**
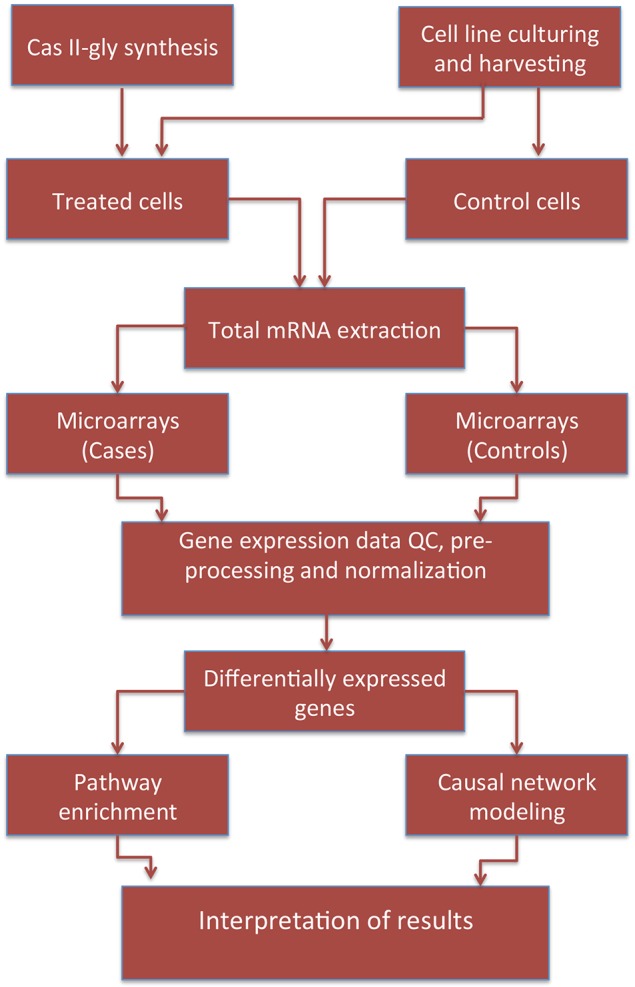
**Pipeline depicting the data/information workflow for this study**.

IKB data is highly-curated since inference procedures are based in two independent sources: First, enrichment scores are determined by hypergeometric tests or Fisher's exact tests—depending on the statistical dependency conditions on the variables under consideration—that measure the overlap between observed and predicted gene sets. Second, IPA calculates Z-scores to evaluate the match between observed and predicted up/down regulation patterns allowing for Bayesian scoring of the results (Krämer et al., 2013). As we mentioned above, gene expression data has been publicly archived at NCBI GEO under accession key GSE41827. For the causal network analysis we used these same data. To consider a differentially expressed gene in this analysis, we used a log Fold Change (LFC) of 1.5 and a corrected *p*-value of 10^−4^. The pipeline followed to perform this work is depicted in Figure 1.

### Overall picture

Taking into account the aforementioned heterogeneous nature of cancer and the promising action of Cas II-gly in other systems, it results important to understand more general mechanisms of action of these chemotherapeutics from the point of view of systems biology. In this work, we focused on pathway analysis—based on our previously published microarray expression data from HeLa cell lines—to observe the signaling pathways that become overexpressed and underexpressed after the action of Cas II-gly, in order to broaden our knowledge of the effect of copper-based therapies. We found that the mechanisms activated by the action of Cas II-gly are related to metal ion response, transcription factor networks, and immune system pathways. On the other hand, the inhibited pathways are related to cell cycle progression, transformation of cells and migration. An important feature that appears to be a consequence of the chemotherapeutic is that some molecules involved in liver protection are overexpressed, suggesting an hepatoprotective action of Cas II-gly. With this approach we remark the necessity of experimental procedures that confirm the results obtained here.

## Results

### Effects on general biological processes

**Figure 2:**
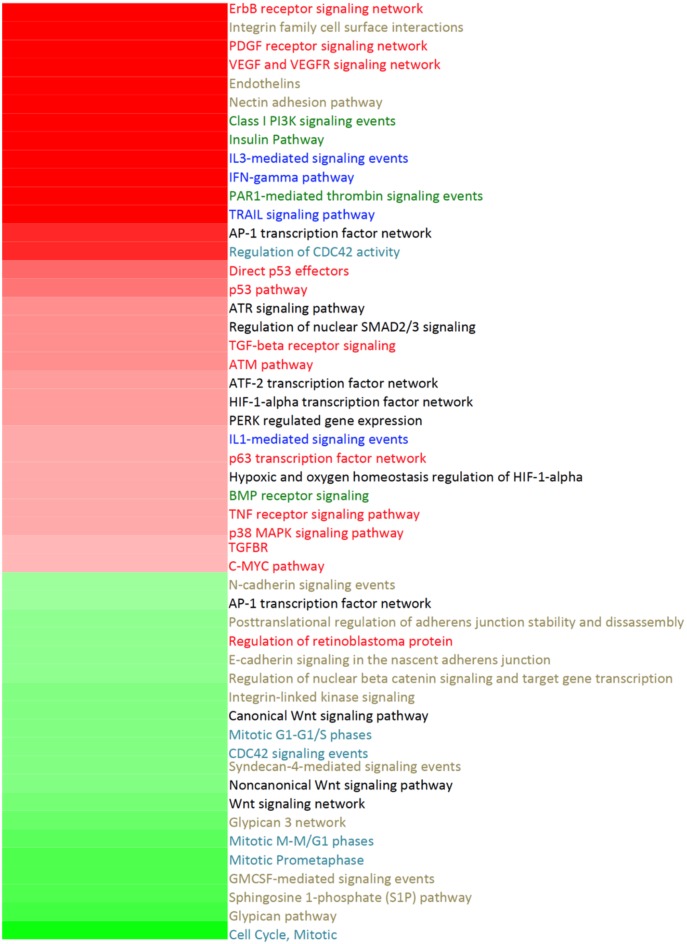
**Heatmap of the main pathways affected by treatment of HeLa Cells with Cas II-gly**. Color gradient corresponds to the −log[*corrected p*−*value*] of the hypergeometric test performed for the pathway enrichment analysis for a *p* = 10^−4^. Overexpressed pathways are shown in red, whereas the underexpressed ones are shown in green. The names of the pathways are colored according to the category which they belong to: oncogene pathways (red), immune system (blue), signal transduction (green), transcription factor networks (black), cell cycle and cell division (turquoise), and cellular structure maintenance (gold names). Please notice that the most general features of the overexpressed pathways are related to oncogene pathways, immune response, signal transduction, and transcription factor networks. Meanwhile the underexpressed pathways are related to cell cycle, cell division and cell structure maintenance.

In Figure 2, the main pathways affected by treatment with Cas II-gly in HeLa cell lines are depicted in the form of a heatmap. Looking at the general features of the overexpressed (red) and underexpressed (green) pathways, we can observe that the processes that are upregulated are mostly related to oncogene pathways (names colored in red in the Figure 2), immune system (blue), signal transduction (green), and transcription factor networks (black). These results indicate that treatment with Cas II-gly could be activating immune response and cell repairing mechanisms in the treated cells. On the other hand, several biological processes which are downregulated are involved in cell cycle (turquoise) as well as cell division; furthermore, several processes related to cellular structural maintenance are also underexpressed (gold labels). This could be indicative of a decrease in the mechanisms of proliferation. Both responses are, in some sense, to be expected after chemotherapeutic action.

**Figure 3:**
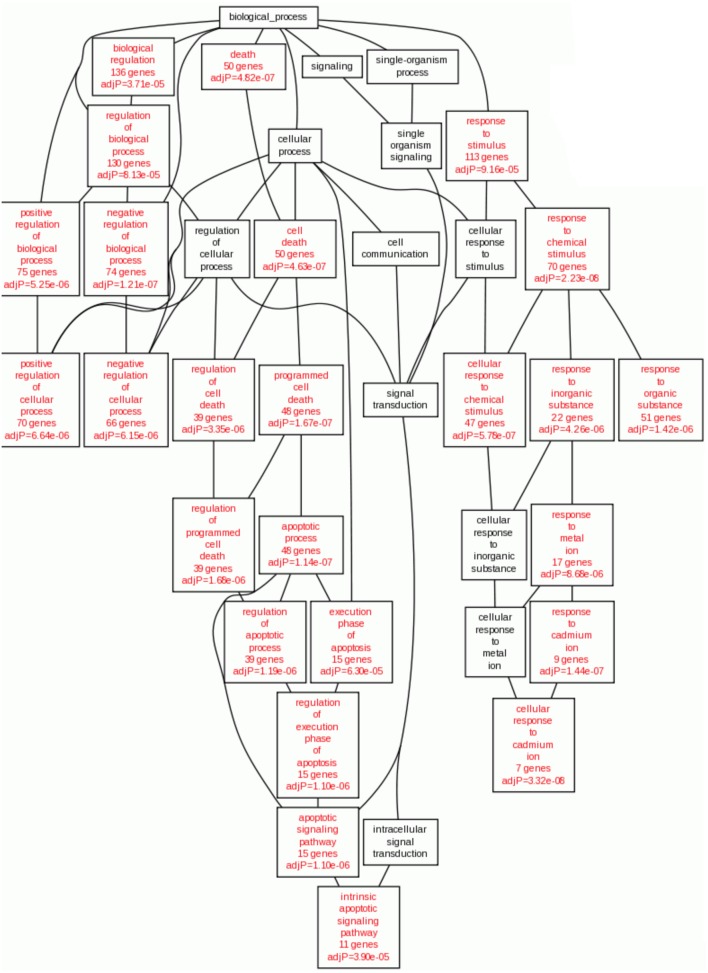
**Statistically enriched GO Biological processes enriched in overexpressed genes in Cas II-gly treated HeLa cells vs. controls**. Statistically significant GO:terms are shown in red (*p* < 10^−5^). It results interesting the fact that the enriched processes with the overexpressed genes are those related to apoptosis and metal ion cellular response.

In the case of Gene Ontology, taking into account the overexpressed genes of the HeLa cells with Cas II-gly treatment, we observe the following: the majority of enriched categories in the Biological Process branch are related to apoptotic processes (Figure 3). Concomitantly with this, there is a branch of processes involved in the response to metal ions. This is consistent with the fact that Cas II-gly is a copper-based therapy and then, the cellular mechanisms to metabolize metal ions are thus activated.

**Figure 4:**
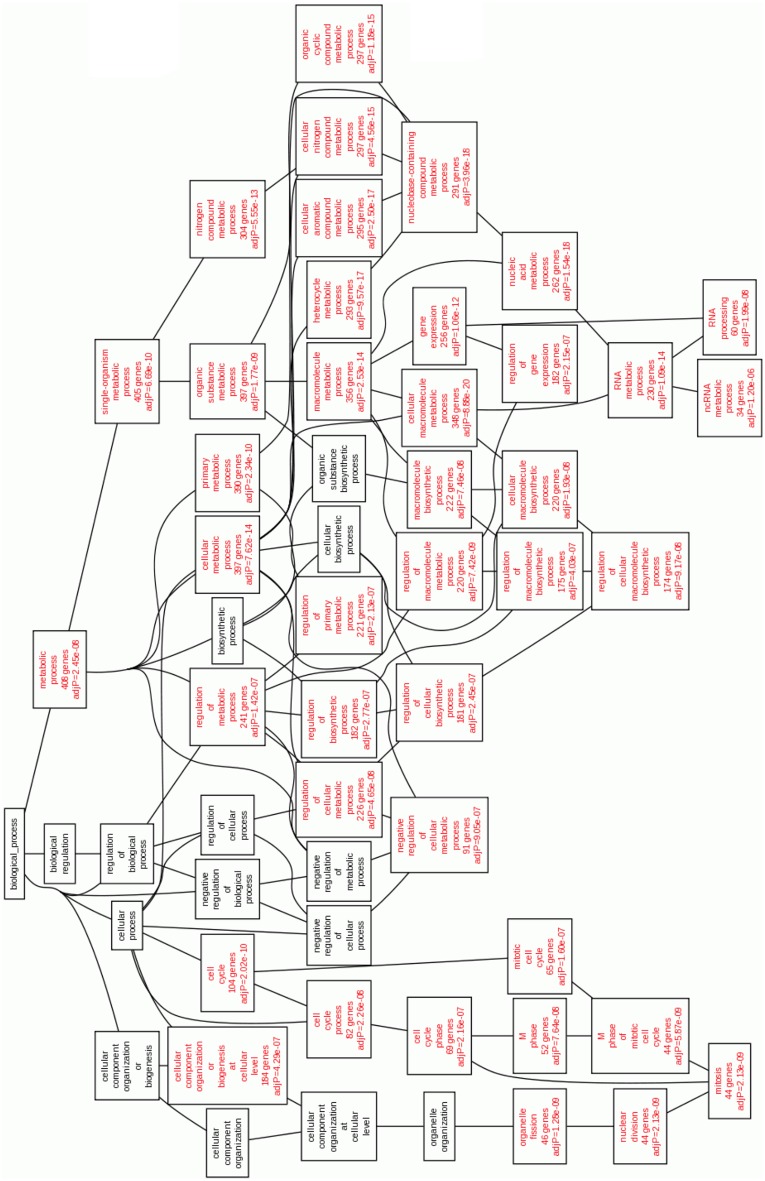
**Statistically enriched GO Biological processes enriched in underexpressed genes in Cas II-gly treated HeLa cells vs. controls**. The enriched processes of the deepest levels are involved in cell division and regulation of transcription.

If we look at the underexpressed genes (Figure 4), the enriched categories (*p* < 10^−5^) of GO Biological Process are related to mitosis and regulation of the transcription processes. Again, this indicates a diminishing of the mitotic and transcriptional events in the Cas II-gly treated cell lines, a positive outcome of the systemic effect of Cas II-gly, as compared with platinum-based cytotoxic drugs.

### Effects on cell cycle regulation

Regarding the regulatory effects of cell cycle processes, causal network analysis shows the following results:

#### Cas II-gly inhibits estrogen-mediated cell cycle progression

**Figure 5:**
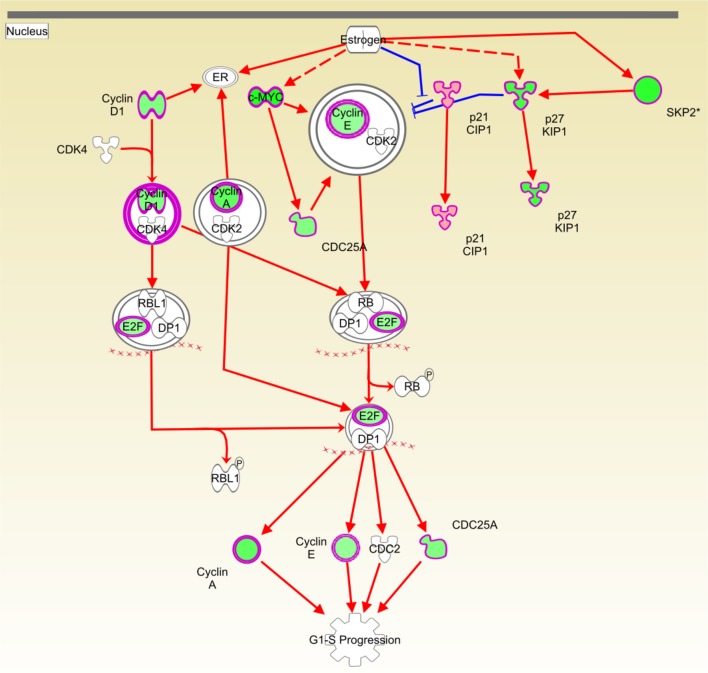
**Causal network depicting the inhibitory effect of Cas II gly on estrogen-mediated G1/S cell cycle progression**. Molecules depicted in green are underexpressed in the whole genome microarray gene expression analysis. Red arrows indicate activation and the blue ones represent inhibition. The original network is provided as a Figure S1.

Figure 5 shows the IKB *estrogen-mediated signaling S-phase entry* pathway. Molecules depicted in green are underexpressed, whereas overexpressed molecules are represented in red. White molecules are not differentially expressed (at the chosen level of statistical significance) with respect to the untreated group. It is clear that the majority of differentially expressed molecules are underexpressed, which indicates a considerable depletion of the process. It is remarkable that estrogen is not differentially expressed; the activation of the pathway could however be triggered via a cross-talk event, in which estrogen downstream molecules could be inhibited by messengers belonging to other pathways. Due to these crosstalk events, the expression level of the estrogen-receptor (ER) may not determine the resulting effect on the pathway, since other components of this pathway could be modulated by any other crosstalking pathways. For instance, in de Anda-Jáuregui et al. (2015), it is shown that the estrogen signaling pathway (ESP) might be affected via the apoptosis signaling pathway in breast cancer samples; this result is consistent with previous experiment of crosstalk between apoptosis and ESP (Clarke et al., 2012; Cook et al., 2014).

#### Cas II-gly downregulates transformation processes in fibroblasts

**Figure 6:**
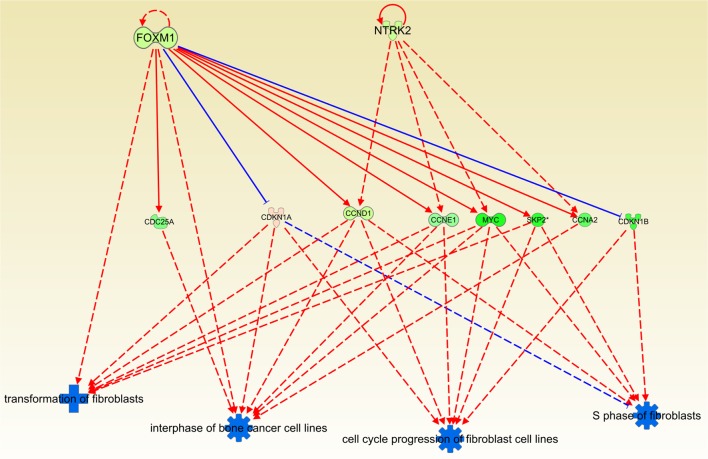
**Causal network depicting the inhibitory effect of Cas II-gly on the ***cell cycle progression of fibroblasts*** and their ***transformation*** processes**. This figure was obtained by using the regulator effects tool in the Ingenuity platform. Blue symbols indicate a predicted inhibition of the associated process. Orange symbols (not shown here) represent a predicted activation. Green molecules symbolize underexpression in the case *versus* the control samples. Analogously, red-pink molecules represent overexpression. It can be observed that the four processes in the bottom part are clearly downregulated, indicating another instance of the effect that Cas II-gly exerts on the arrest of the cell cycle. The original figure, containing all molecules and associated processes, is provided as Figure S2. For clarity, we removed those molecules and arrows which are not directly linked to those processes.

An *upstream regulator analysis* from IPA knowledge base, shows that FOXM1 and NTRK2 control the processes of proliferation of fibroblasts, being these effects proportional to their expression level. The lower the expression level in those molecules, the lower the activity of those events. These two molecules promote the activity of several other participants in the *regulation of fibroblast cell cycle and transformation*: CDC25, CDKN1A, etc. (Figure 6). It is worth to mention that *transformation of fibroblasts* is a crucial step in tumor progression since these cells are involved in the formation of extracellular matrix (ECM). Another feature of fibroblast function is their role in mitotic process induced by cellular damage. Thus, downregulation of this process contributes to decrease malignant effects such as tumor growth, invasiveness or vascularization.

### Cas II-gly treatment reduces cell migration in HeLa cell lines

**Figure 7:**
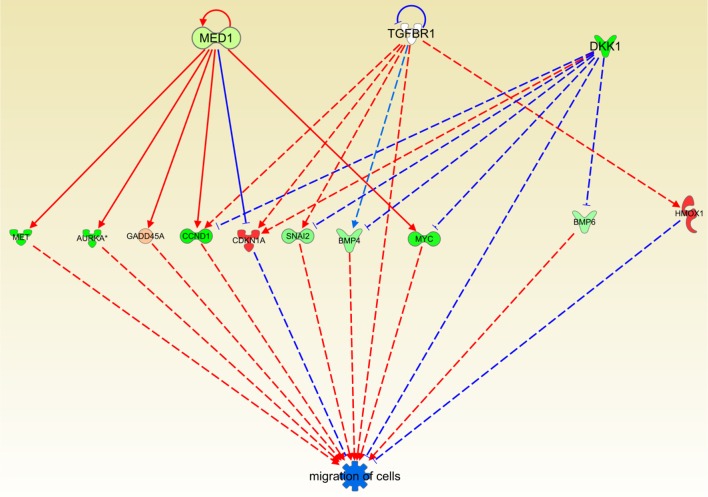
**Causal network depicting the effect inhibitory of Cas II-gly on cell migration processes**. The color code is the same than the previous figure. Notice that several molecules in the figure are underexpressed and those molecules, being underexpressed, their activator role is dampened. At the same time, overexpressed molecules CDKN1A and HMOX1 inhibit the migration process. As in the previous figure, the original one is provided as Figure S3.

It is already known that MED1, TGFBR1 and DKK1 underexpression decreases *migration of cells*, (Larsson et al., 2001; Ohlmann et al., 2010; Cui et al., 2012; Li et al., 2013). In the IPA knowledge base analysis it is shown that those molecules are underexpressed which in turn diminish the migration of cells (Figure 7). Again, the effect of Cas II-gly results in the inhibition of a very well-known hallmark of cancer: uncontrolled cell migration (Hanahan and Weinberg, 2000, 2011). The result presented here is another instance of the lowering of cell migration by the action of a cytotoxic agent.

### Effects on liver injury and protection

**Figure 8:**
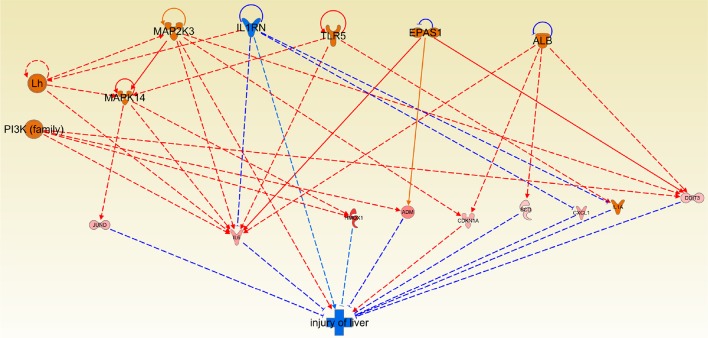
**Causal network depicting the effect of Cas II-gly on liver injury and protection**. Please notice how the activating arrows are decreasing the effect of liver damage, thus indicating that Cas II-gly treatment exerts a hepatoprotective effect. The original figure obtained by the regulator effect tool of Ingenuity platform is provided as Figure S4.

One of the most evident and undesirable side effects of chemotherapy is the one related to hepatotoxicity (Kufe et al., 2003; Koschny et al., 2007), since it affects at organismal level and very often is even more harmful than the tumor itself. In this regard, we examined the effect of Cas II-gly treatment on the *liver damage* pathway in HeLa cell lines. We obtained a promising result: The mechanisms of protection to the liver are activated and the damage of liver tissue mechanisms are also arrested (Figure 8).

It is worth to mention that there are several findings that support this assertion: For instance, in Wang et al. (2011) it is shown that IL6 decreases injury of liver. Since this protein is overexpressed in our HeLa samples, we can argue that this mechanism could be present under exposure of Cas II-gly. Furthermore, Jund depletion increases injury of liver (Marden et al., 2008). Due to the fact that Jund is overexpressed in our samples, this could also be indicative that there is another mechanism of liver protection. Participation of MAPK14 could be relevant in the context of liver protection. This molecule is not differentially expressed in our samples, however, its target molecules were differentially expressed.

Another instance of molecules related to mechanisms of liver protection is heme-oxygenase 1, HMOX1. Quite remarkably, HMOX1 protects liver in several forms (Seki and Brenner, 2008; Sass et al., 2012). Moreover, HMOX1 is the third most overexpressed gene in our samples (Table 1); hence, to argue that this molecule is importantly influencing the mechanisms of liver protection is reasonable. Li et al. (2009) demonstrate that knock-out mice of Scd1 gene increase liver damage. Overexpression of this molecule in our samples could be indicative of other liver protecting mechanisms.

**Table 1:** **Top20 of differentially expressed genes in HeLa cell lines under the effect of Cas II-gly**.

**Ratio**	**ID**	**Entrez gene name**
6.92	HSPA6	Heat shock 70kDa protein 6 (HSP70B′)
4.41	IL8	Chemokine (C-X-C motif) ligand 8
4.16	HMOX1	Heme oxygenase 1
4.1	MAFF	V-maf avian musculoaponeurotic fibrosarcoma oncogene homolog F
3.74	ATF3	Activating transcription factor 3
3.09	SLC30A1	Solute carrier family 30 (zinc transporter), member 1
2.94	GADD45B	Growth arrest and DNA-damage-inducible, beta
2.75	C11orf96	Chromosome 11 open reading frame 96
2.54	DNAJB1	DnaJ (Hsp40) homolog, subfamily B, member 1
2.53	ERRFI1	ERBB receptor feedback inhibitor 1
−2.41	C14orf104	Dynein, axonemal, assembly factor 2
−2.43	GEMIN4	Gem (nuclear organelle) associated protein 4
−2.49	SKP2	S-phase kinase-associated protein 2, E3 ubiquitin protein ligase
−2.51	FZD2	Frizzled class receptor 2
−2.66	FADD	Fas (TNFRSF6)-associated via death domain
−2.66	GAS1	Growth arrest-specific 1
−2.67	FBXO5	F-box protein 5
−2.73	MYO10	Myosin X
−3.2	MYC	V-myc avian myelocytomatosis viral oncogene homolog
−3.52	DKK1	Dickkopf WNT signaling pathway inhibitor 1

## Discussion

Cancer is a complex disease, with a plethora of intricate mechanisms that determine the features of specific phenotypes which in turn give place to distinct prognosis and treatment. Chemotherapy is one of the most used treatments to face this condition. Casiopeínas have shown an important chemotherapeutic effect on cancer cell lines (Valencia-Cruz et al., 2013). The intrinsic route to apoptosis is favored by caspase-3 activation via cyt C overexpression, high ROS concentrations, as well as cell cycle arrest; whereas the extrinsic route to apoptosis is somehow diminished due to very low expression of procaspase-8 and caspase-8. Antiapoptotic processes are switched-down, due to low levels of Bcl-2. Furthermore, copper-based therapy could be more convenient than other cytotoxic agents in terms of a decrease in side effects, since copper, being an essential trace element can be metabolized. Casiopeínas reach the membrane of mitochondria and exerts a pro-apoptotic effect (Valencia-Cruz et al., 2013), as can be seen in Figure 3. As it was previously mentioned, Cas II-gly activates molecules that participate in oxidative stress processes and reduces cell viability by the intrinsic apoptotic pathway. In this work, we developed an integrative systems biology approach to elucidate the effect of Cas II-gly treatment in HeLa cell lines, observing new features, namely, *inhibition of G1/S transition phase*, decrease of *cell transformation process* and a decrease of cell migration.

Cas II-gly promotes *apoptosis* and *metal ion signaling* responses (Valencia-Cruz et al., 2013). At the same time, with the results shown by this analysis, we demonstrate that Cas II-gly inhibits cell proliferation, cell migration, transformation of cells as well as cell cycle progression in HeLa cell lines. Furthermore, this treatment promotes the liver repairing system as well as the activation of molecules related to decrease of liver damage.

Regarding the inhibitory effect that Cas II-gly has on cell cycle and transcription, it can be observed in Figures 5–7, that the inducer molecules involved in those processes are underexpressed. A good example of this is depicted in Figure 5, where a considerable subset of the molecules involved in the *estrogen-mediated G1/S phase* progression are underexpressed. A similar effect is observed in Figure 6, where the upstream molecules FOXM1 and NTRK2 are downregulated. This downregulation event affects the cell-division molecules CDC25A, CCND1, CCNE1, MYC, SKP2, and CCNA2. Interestingly, FOXM1 underexpression abolishes the negative feedback with CDKN1A (Wang et al., 2002, 2007); this cyclin-dependent kinase inhibits the *S-phase entry of fibroblasts* (Sherr and Roberts, 1999). It is worth to mention that, despite the fact that the network depicted in Figure 6 corresponds to processes diminished in fibroblasts and bone cancer cell lines, the molecules involved in these processes are ubiquitous in all cell types. Furthermore, some of these molecules are known to be markers of tumor growth as well as cell cycle progression. As it was explained before, Ingenuity Pathway Analysis contains a large database of interactions. Those interactions have been highly curated by literature or *ex professo* experiments. The observed processes of Figure 6 correspond to interactions of those molecules observed on those cell types. However, the interactions are also common in many other cell types. Based on that, to extrapolate the effects that Cas-II gly exerts on fibroblasts to HeLa cell lines may be appropriate in terms of the set of molecules implied in those processes.

By observing Figure 7, it can be noticed that MED1 exerts a repression on CDKN1A (p21) which in turn inhibits cell migration (Lee et al., 2013); the same is true of HMOX1 (Lu et al., 2012); thus, concomitant overexpression of these molecules exert a negative role in migration. Other molecules involved in the process of cell migration are underexpressed; all of those molecules have a pro-migration role. Hence, their underexpression generates a global anti-migration effect. This could be relevant in terms of the complementary role that Casiopeínas may have as a therapeutic treatment: Casiopeínas act as a cytotoxic (Bravo-Gómez et al., 2009; Gutiérrez et al., 2013; Valencia-Cruz et al., 2013) and concomitantly they may exert an inhibitory effect on cell migration.

In this work, we have performed pathway analysis with two different but complementary strategies. The general mechanism of action of Cas II-gly is known in terms of the damage to DNA and mitochondrial stress. However, the whole set of different effects that this drug exerts on the cell is not well-known. With the analysis performed here, we observe that some molecules that have been related to liver damage and repairing are differentially expressed in the treated cells respecting the control ones. These molecules are ubiquitous in any cell type: JUND, IL6, HMOX1, ADM, MAPK14, DDT3. The importance of these results lies on the fact that these molecules are consistently associated with protection from hepatic damage (at the systemic level). The experiment shown here, was performed in a cervical cancer cell line. However, the effect of Cas II-gly in this cell line activates (or deactivates) specific molecules that have been observed to perform a particular effect at the systemic level. It remains to be tested (by means of *in vitro* but even more importantly, by *in vivo* models and clinical experiments) whether the molecular pathways activated by Cas II-gly are actually able to induce such liver protecting processes in living subjects.

Cas II-gly is a potent and promising chemotherapy. In this regard, the results presented here corroborate the potential usage of this drug as an anticancer therapy. One of the most relevant results is related to liver damage. In Figure 8 can be observed that almost every molecule in the figure is overexpressed, and these molecules inhibit liver damage. Activation of molecules downstream of MAP2K3 allows a protection of the liver, with the exception of p21 (CDKN1A). The overall effect of those interactions is hepatoprotective. The last result might be helpful in terms of the clinical potential, given the fact that the majority of metal ion therapy is based on platinum, and it is also well-known that platinum cannot be naturally metabolized. Furthermore, the GO (biological processes) terms in the overexpressed genes sets are enriched for the response of the *metal ion signaling pathway*, reflecting the fact that cells are facing to this external agent.

Further steps toward a complete understanding of the mechanisms of action of Casiopeínas, involve a comparative analysis *in vivo*. Experiments in this regard are currently on their way. Such analyses, as well as a toxicological study (phase I clinical trial) in human subjects are ongoing within our group. In the meantime, the systems biology approach presented here, shows general and also specific features of the regulatory effect that Cas II-gly may exert, and this knowledge could be relevant to provide a more integrated overview of the processes in which novel anticancer therapies are involved, looking for the understanding of global chemotherapeutic mechanisms.

## Author contributions

JE performed analysis and calculations, contributed with algorithms and analytical methods, collaborated in the discussion, collaborated in writing the manuscript, EH conceived and designed the study, contributed with algorithms and analytical methods, performed analysis and calculations, collaborated in the discussion, collaborated in writing the manuscript. CM performed the experiments, collaborated in the discussion, collaborated in writing the manuscript. LR synthesized the drugs, collaborated in the discussion, collaborated in writing the manuscript. All authors read and approved the final manuscript.

## Funding

This work was supported by CONACYT (grant no. 179431/2012), as well as by federal funding from the National Institute of Genomic Medicine (Mexico). Additional support has been granted by the National Laboratory of Complexity Sciences (grant no. 232647/2014 CONACYT).

### Conflict of interest statement

The authors declare that the research was conducted in the absence of any commercial or financial relationships that could be construed as a potential conflict of interest.
