# Supplementary figures and images for: Network Analysis Shows Novel Molecular Mechanisms of Action for Copper-Based Chemotherapy
Source: Front Physiol. 2016 Jan 12;6:406. doi: 10.3389/fphys.2015.00406 (PMC4709449; doi:10.3389/fphys.2015.00406)

Nucleus

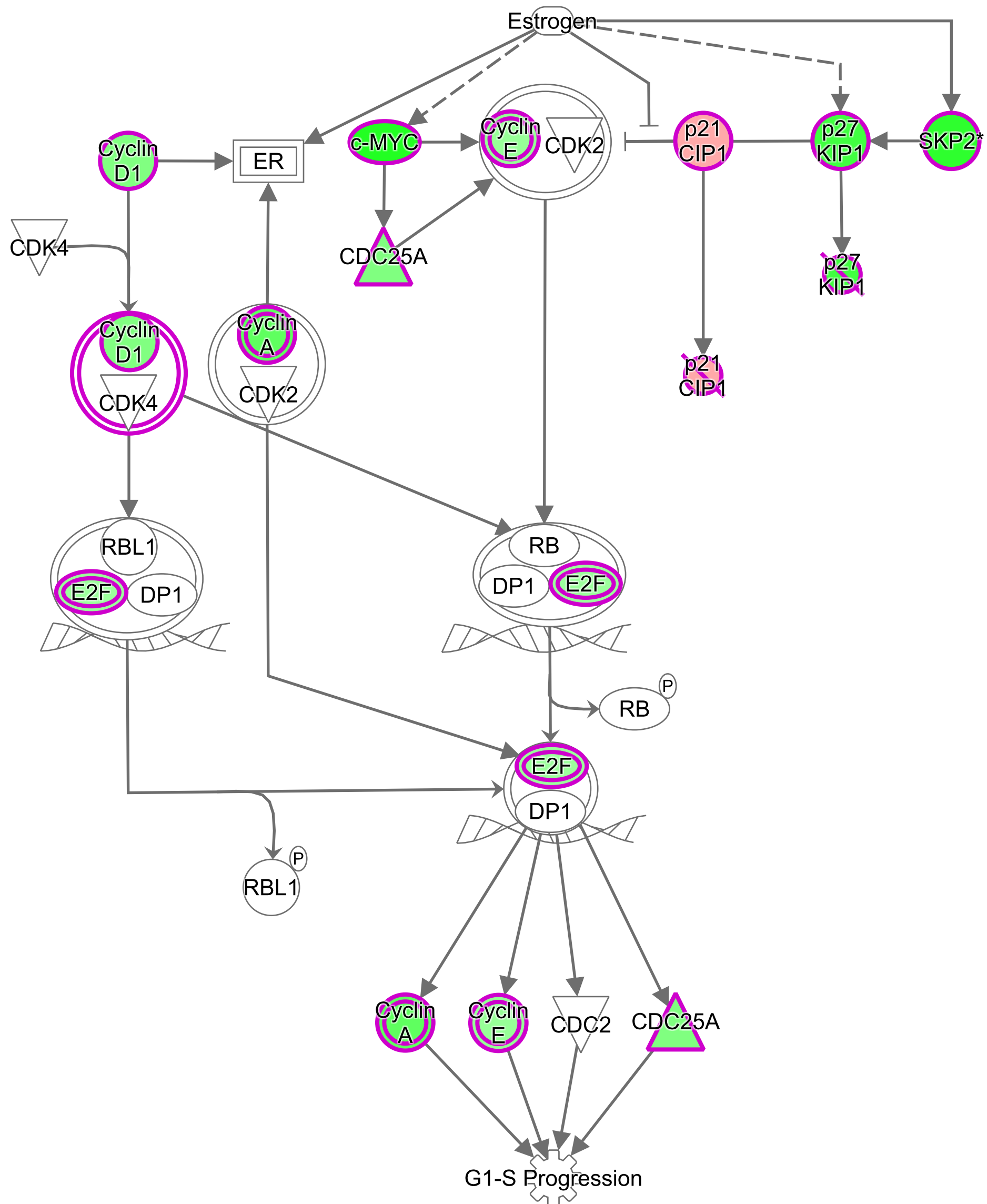

Supplement: Supplementary file 1 [file Image1.PDF]

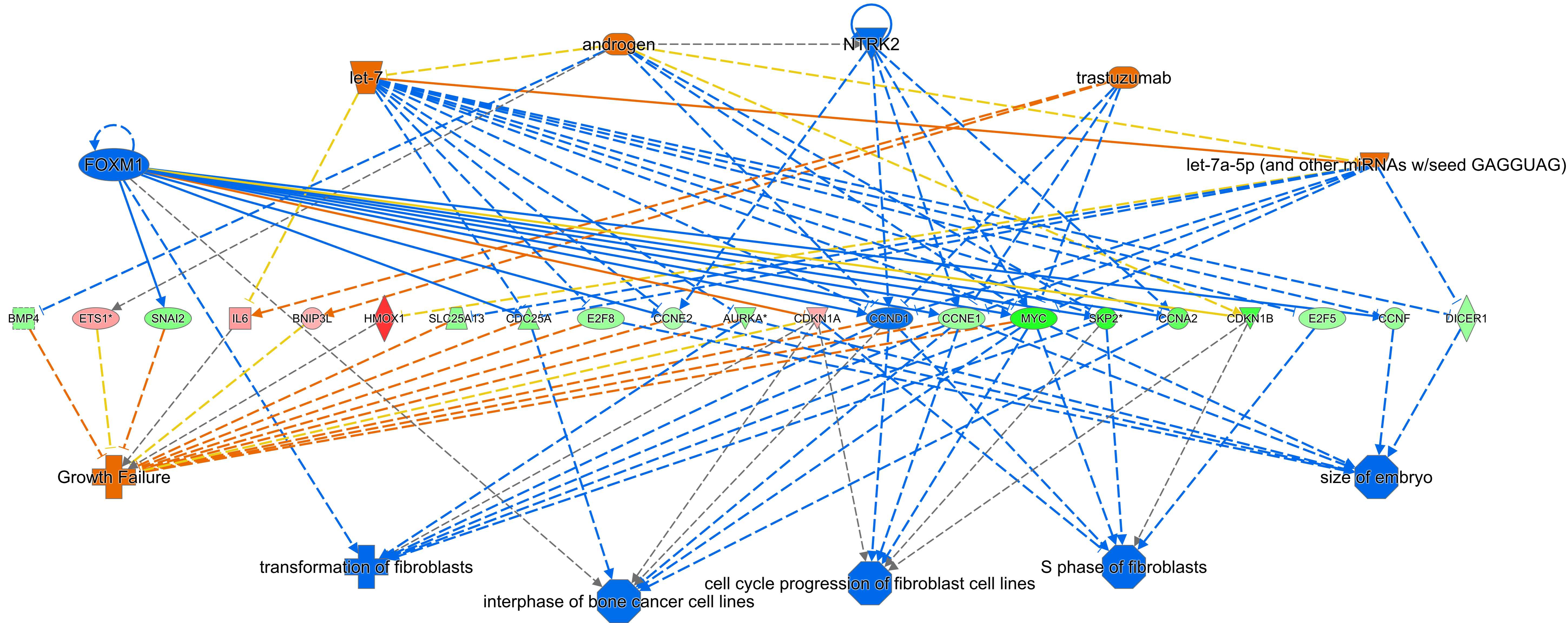

Supplement: Supplementary file 2 [file Image2.PDF]

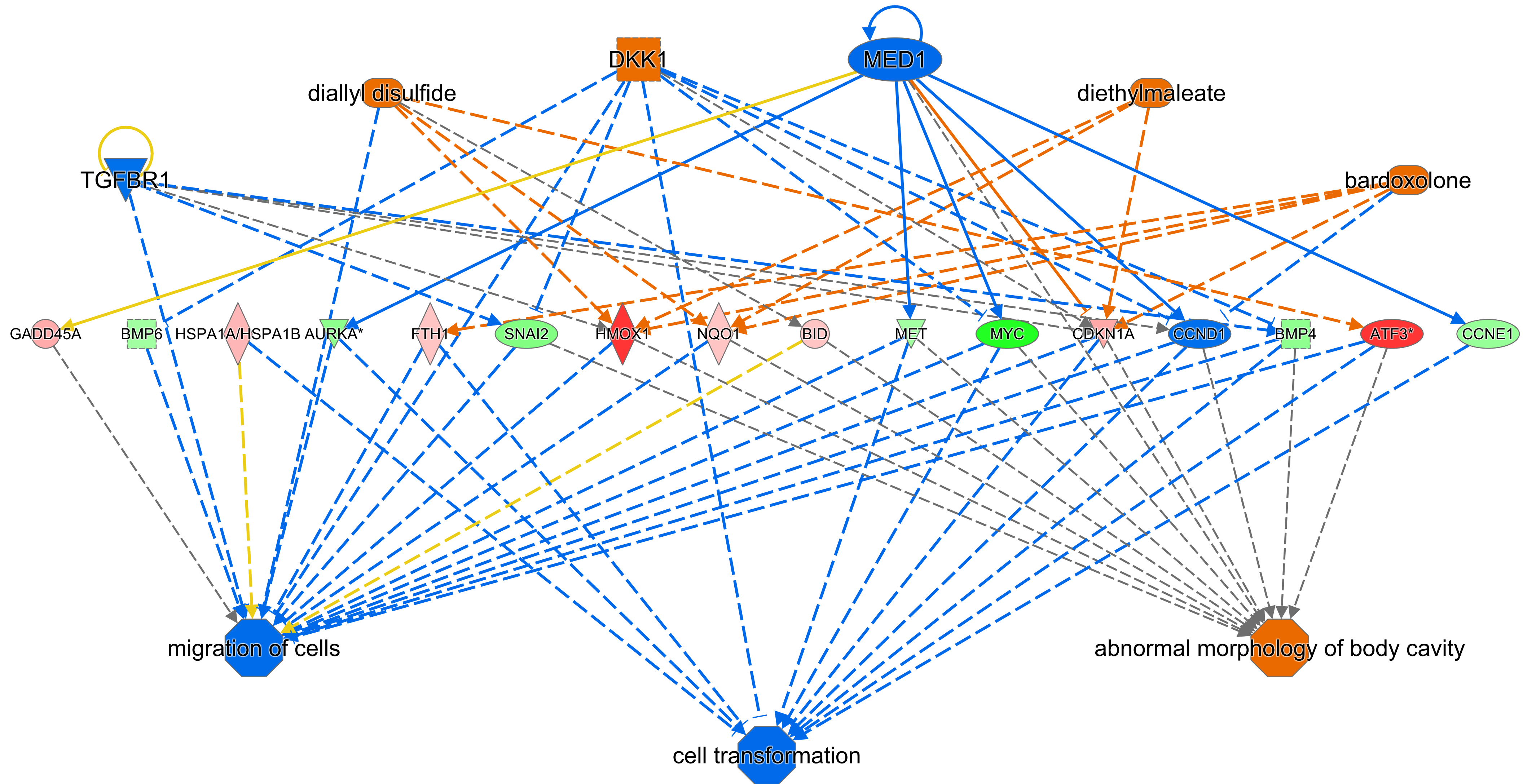

Supplement: Supplementary file 3 [file Image3.PDF]

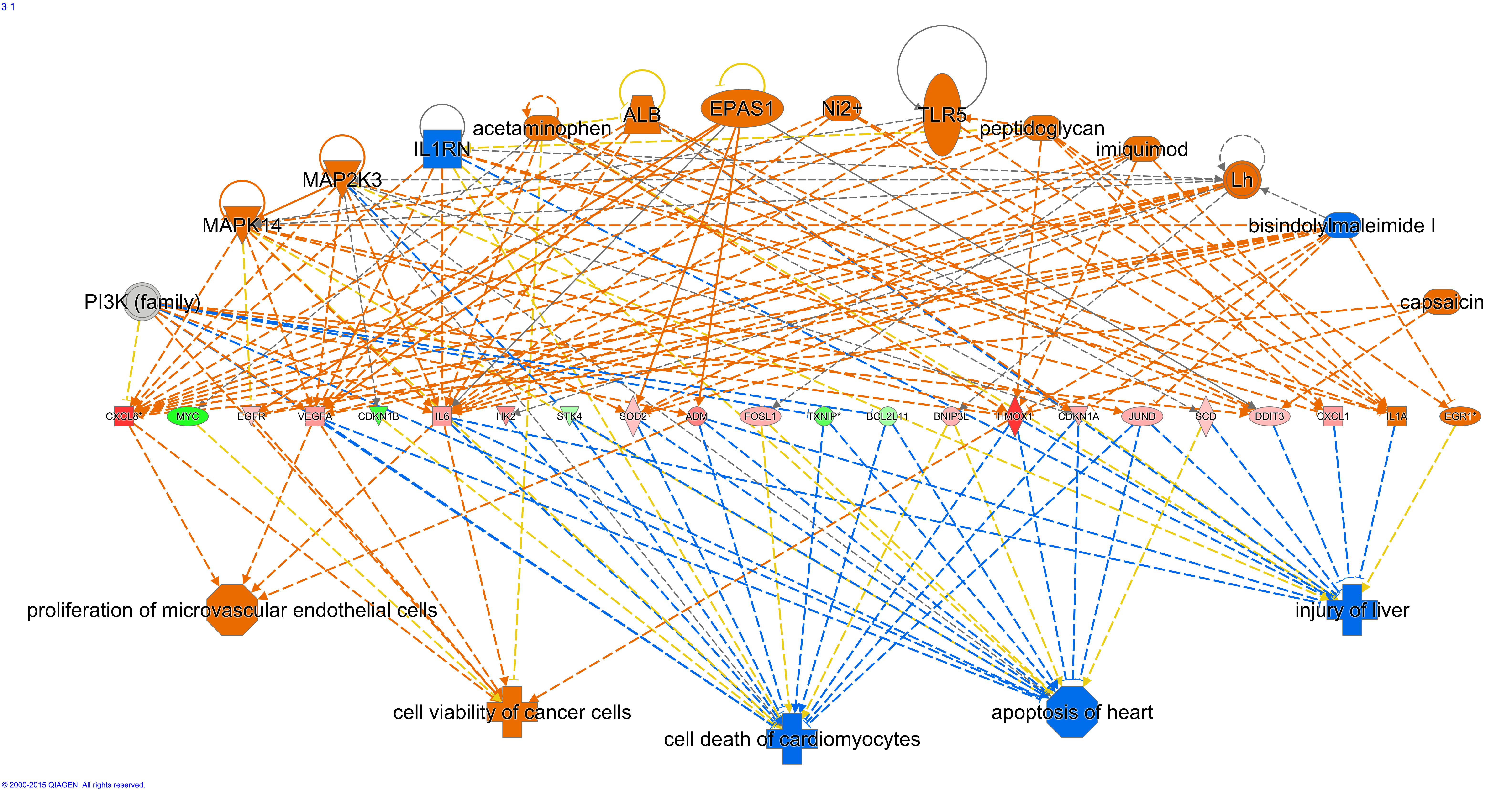

Supplement: Supplementary file 4 [file Image4.PDF]
